# Novel probiotic approach to counter *Paenibacillus larvae* infection in honey bees

**DOI:** 10.1038/s41396-019-0541-6

**Published:** 2019-10-29

**Authors:** Brendan A. Daisley, Andrew P. Pitek, John A. Chmiel, Kait F. Al, Anna M. Chernyshova, Kyrillos M. Faragalla, Jeremy P. Burton, Graham J. Thompson, Gregor Reid

**Affiliations:** 1 Centre for Human Microbiome and Probiotic Research, Lawson Health Research Institute, London, ON Canada; 2 Department of Microbiology and Immunology, The University of Western Ontario, London, ON Canada; 3 Department of Biology, The University of Western Ontario, London, ON Canada; 4 Department of Surgery, The University of Western Ontario, London, ON Canada

**Keywords:** Microbiome, Microbial ecology, Applied microbiology, Conservation biology

## Abstract

American foulbrood (AFB) is a highly virulent disease afflicting honey bees (*Apis mellifera*). The causative organism, *Paenibacillus larvae*, attacks honey bee brood and renders entire hives dysfunctional during active disease states, but more commonly resides in hives asymptomatically as inactive spores that elude even vigilant beekeepers. The mechanism of this pathogenic transition is not fully understood, and no cure exists for AFB. Here, we evaluated how hive supplementation with probiotic lactobacilli (delivered through a nutrient patty; BioPatty) affected colony resistance towards a naturally occurring AFB outbreak. Results demonstrated a significantly lower pathogen load and proteolytic activity of honey bee larvae from BioPatty-treated hives. Interestingly, a distinctive shift in the microbiota composition of adult nurse bees occurred irrespective of treatment group during the monitoring period, but only vehicle-supplemented nurse bees exhibited higher *P. larvae* loads. In vitro experiments utilizing laboratory-reared honey bee larvae showed *Lactobacillus plantarum* Lp39, *Lactobacillus rhamnosus* GR-1, and *Lactobacillus kunkeei* BR-1 (contained in the BioPatty) could reduce pathogen load, upregulate expression of key immune genes, and improve survival during *P. larvae* infection. These findings suggest the usage of a lactobacilli-containing hive supplement, which is practical and affordable for beekeepers, may be effective for reducing enzootic pathogen-related hive losses.

## Introduction

Managed honey bees (*Apis mellifera*) perform critical pollination services to many agricultural crops and contribute an estimated $225 billion USD annually to the global economy [1]. However, the health of this insect species is an ongoing concern, as illustrated by persistent population decline over the last decade [2–4]. The causal factors precipitating this decline likely include a combination of pesticide exposure, infectious disease, and loss of habitat [5].

One well-known pathogen afflicting honey bee brood is the spore-forming bacterium *Paenibacillus larvae*, which causes American foulbrood (AFB). This highly adapted pathogen infects *A. mellifera* during early development and can kill brood through secretion of secondary metabolites (that have antimicrobial properties to counter microbial competitors) and chitin-degrading enzymes (enabling degradation of the peritrophic matrix) that allow breaching of the midgut epithelium, invasion of the haemocoel, and decomposition of the larva to a ropy mass [6]. ERIC I isolates of *P. larvae* are most common and predicted to produce more secondary metabolites and fewer virulence factors with a 100% lethality index of 10–12 days, whereas ERIC II–IV isolates require only 6–7 days to kill infected honey bee larvae [7, 8].

AFB is a notifiable disease in many countries and requires the destruction of clinically infected hives [9]. This is because attempts at hive rescue are outweighed by the extreme contagiousness and subsequent risk of disease spread to surrounding apiaries and to wild pollinators [10]. Despite its nearly cosmopolitan distribution and enzootic state in most honey bee hives [11], this pathogen often remains dormant in its spore-form and does not induce manifestations of AFB [12]. It has been suggested that *P. larvae* may exist as a pathobiont in the native microbiota of adult worker bees, from where it is passively and constitutively transmitted throughout the hive to fresh brood cells [12].

Measures to control AFB in apiaries include antibiotic treatment [13], selective breeding for hygienic behaviour [14], application of bioactive essential oils [15], bacteriophage therapy [16], and administration of synthetic indoles to inhibit germination of *P. larvae* [17]. These disease management approaches are helpful but often ineffective, and hives remain vulnerable to AFB. One alternative being considered is the supplementation of colonies with beneficial bacteria such as *Lactobacillus* spp. [18]. Findings from model systems support this approach, showing that *Lactobacillus plantarum* Lp39 can improve the innate immune response and resistance towards opportunistic infection in *Drosophila melanogaster* [19]. A reduction of pesticide toxicity via *Lactobacillus rhamnosus* GR-1 supplementation in *D. melanogaster* [20] and protection against harmful microorganisms by various *Lactobacillus* and *Bifidobacterium* spp. [21–24] has also been reported, suggesting these beneficial bacterial may be useful for directly addressing some of the causal factors implicated in honey bee decline. Additionally, resistance factors that arises through antibiotic administration are less likely to evolve with probiotic application [25]. Long-lasting benefits to honey bee longevity have been observed following relatively short probiotic supplementation periods and without the necessity of host colonization—demonstrating an intermittent dosing schedule, which also reduced hive disturbance, is favourable [26].

In this study, we capitalized on a naturally occurring AFB outbreak to test a lactobacilli-infused nutrient patty (referred to as the BioPatty) for its ability to suppress *P. larvae* under normal field conditions. Signs of AFB developed in the experimental apiary within a few weeks as expected, providing an opportunity to examine the pathological microbial shifts that occur during disease progression and to quantitatively assess the effect of BioPatty supplementation on hive health.

## Methods

### Bacterial strains and cultures

*Lactobacillus plantarum* Lp39 (Lp39; American Type Culture Collect (ATCC), number 14917), *Lactobacillus rhamnosus* GR-1 (LGR-1; ATCC number 55826), and *Lactobacillus kunkeei* BR-1 (LkBR-1; previously isolated from a healthy honey bee hive) were routinely cultured anaerobically at 37 °C using de Man, Rogosa, and Sharpe (catalogue number 288130; BD Difco) broth or agar supplemented with 10 g/L d-fructose (catalogue number F-3510; Sigma-Aldrich; MRS-F), unless otherwise stated. Isolated *P. larvae* BMR43-81 (from diseased honey bee larvae in this study) was routinely cultured in a microaerophilic incubator at 37 °C under 5% CO_2_ using modified Mueller–Hinton (2 g/L Mueller–Hinton broth (catalogue number 212322; BD Difco) and 15 g/L yeast extract (catalogue number 212750; BD Difco); MY) broth and agar, unless otherwise stated. Honey bee isolates *Enterobacter hormaechei* B0003, *Paenibacillus illinoisensis* B0004, *Hafnia paralvei* B0008, and *Lactobacillus apis* B0011 used for inhibition assay experiments were from a geographically distinct honey bee hive exhibiting no signs of disease. Isolates *Enterobacter hormaechei* B0003, *Paenibacillus illinoisensis* B0004, *Hafnia paralvei* B0008 were cultured aerobically at 37 °C in MY, whereas *Lactobacillus apis* B0011 was cultured anaerobically at 37 °C in MRS-F.

### Apiary set up, treatment groups, and sampling procedure

Field trials were performed on managed Chilean-sourced honey bees (*A. mellifera*) in an experimental apiary owned and operated through Western University (London, Ontario, Canada) for the purposes of scientific investigation. The apiary consisted of ten colonies located in a single geographic location and housed in standard Langstroth hives that were elevated ~36 inches above ground level using wooden support beams. Two hives, designated ‘hive A’ and ‘hive B’, were used for each of the following treatment groups: (1) a no-treatment control (NTC) group that received equal levels of physical disturbance without any form of supplementation, (2) a vehicle pollen patty group that received nutritional supplementation in the form of a 250-g patty containing standard pollen substitute ingredients (28.5 g of soy flour, 74.1 g of granulated sucrose, 15.4 g of debittered brewer’s yeast, 132.1 g of a 2:1 (w/v) simple sucrose-based syrup solution) with the addition of 4 mL of vehicle (0.01 M phosphate-buffered saline (PBS)) per patty, and (3) a BioPatty group, which received the 250 g of base pollen patty ingredients with the additional infusion of Lp39, LGR-1, and LkBR-1 each at a final concentration of 10 colony-forming units (CFU)/g. Supplementation of hives occurred twice during the field trial on day 0 and day 7. Sampling of hives occurred on days 0 and 12 during which 30 adult nurse bees (located on frames with active brood) were collected from each hive. Sampling of larvae (third- to fifth-instar) occurred only on day 12 as our original intentions were not to monitor early life stages. Individuals were collected equally from each hive per treatment group (i.e. the same number of samples were taken from both ‘hive A’ and ‘hive B’ for each of the three experimental groups). Pooling of samples occurred within the same hive and the same number of pooled samples were collected from each hive per treatment group. Colony ID was recorded but was not considered in downstream analyses in favour of preserving a more robust dataset. Following the detection of AFB on day 12, honey bees were promptly euthanized, and hives scorched according to local regulations. Thus, no follow-up survey could be performed to track further disease progression.

### Isolation and identification of *P. larvae* bacterial colonies

Standard methods for identification of AFB were followed as previously described [27]. Briefly, infected larvae exhibiting signs of active disease were extracted from the hive, homogenized in equal volumes of 0.01 M PBS (w/v) using a sterile motorized pestle, serial diluted and spread plated on MYPGP (10 g/L Mueller–Hinton broth, 15 g/L yeast extract, 3 g/L K_2_HPO_4_, and 1 g/L sodium pyruvate), brain heart infusion (BHI; catalogue number 211059; BD Difco), and MY agar. Isolated *P. larvae* colonies were visually verified on the basis of their Gram-stain and morphological characteristics, and then re-streaked to obtain pure cultures from which DNA was extracted as described previously [28]. Universal 16S rRNA gene primers pA (5′-AGAGTTTGATCCTGGCTCAG-3′) and pH (5′-AAGGAGGTGATCCAGCCGCA-3′) were used for PCR as previously described [28]. The amplified product was then purified by 1.0% agarose gel electrophoresis, extracted with a QIAquick gel extraction kit (catalogue number 28704; Qiagen), and sequenced using the aforementioned primers with the Applied Biosystems 3730 Analyzer platform at the London Regional Genomics Centre (Robarts Research Institute, London, Canada). DNA was similarly extracted from *Enterobacter hormaechei* B0003, *Paenibacillus illinoisensis* B0004, *Hafnia paralvei* B0008, and *Lactobacillus apis* B0011 isolates. The corresponding 16S rRNA partial sequences were uploaded to NCBI GenBank (accession numbers: MK618560 and MK618171–MK618174).

### Repetitive element sequence-based PCR

Briefly, DNA from a single colony of the *P. larvae* isolate was extracted using the InstaGene (Bio-Rad) matrix protocol following manufacturer’s instructions. Genotyping of the *P. larvae* isolate was then performed using the ERIC1R (5′-ATGTAAGCTCCTGGGGATTCAC-3′) and ERIC2 (5′-AAGTAAGTGACTGGGGTGAGCG-3′) primers as previously described [29]. Using 10 µL of the amplified products, banding pattern was analyzed on a 0.8% agarose gel stained with ethidium bromide and visualized under UV light in an AlphaImager 2200 station (Innotech).

### qPCR-based quantification of microbial communities in larval and adult honey bee samples

Honey bee larvae (whole body) and adults (dissected whole abdomens) were surface sterilized using 0.25% sodium hypochlorite, followed by a 30-s wash in ddH_2_O. DNA was then extracted from samples using the previously described CTAB method [30]. Bacterial loads were then determined by qPCR with the Power SYBR Green kit (Applied Biosystems) using universal and phylotype-specific 16S rRNA primers listed in Supplementary Table 1. All qPCR reactions were performed in DNase- and RNase-free 384-well microplates on a Quant Studio 5 Real-Time PCR System (Applied Biosystems) and analyzed with associated software. Copy numbers of target 16S rRNA genes were calculated as previously described using established primer efficiencies and limit of detections [30–33].

### 16S rRNA gene library preparation

Targeted amplification of the 16S rRNA V4 region was performed using the established GOLAY-barcoded primers (5′–3′) ACACTCTTTCCCTACACGACGCTCTTCCGATCTNNNNxxxxxxxxxxxxGTGCCAGCMGCCGCGGTAA and (5′–3′) CGGTCTCGGCATTCCTGCTGAACCGCTCTTCCGATCTNNNNxxxxxxxxxxxxGGACTACHVGGGTWTCTAAT wherein ‘xxxxxxxxxxxx’ represents the sample-specific 12-mer barcode following the Illumina adaptor sequence used for downstream library construction [34]. Utilizing a BioMek Automated Workstation (Beckman Coulter), 2 µL of sample DNA (5 ng/µL) was added to a 96-well 0.2-mL PCR plate containing 10 µL of each primer per well (3.2 pmol/µL), followed by the addition of 20 µL of GoTaq 2X Colourless Master Mix (Promega). Final plates were then sealed using PCR-grade adhesive aluminium foil and placed in a Prime Thermal Cycler (Technie). PCR reaction conditions were as follows: an initial activation step at 95 °C, followed by 25 cycles of 95 °C for 1 min, 52 °C for 1 min, and 72 °C for 1 min. After completion, the thermocycler was held at 4 °C, and amplicons subsequently stored at −20 °C until further processing.

### 16S rRNA sequencing and data analysis

Processing of amplicon libraries was conducted at the London Regional Genomics Centre (Robarts Research Institute, London, Canada) in which amplicons were quantified using PicoGreen (Quant-It; Life Technologies, Burlington, ON), pooled at equimolar ratios, and sequenced on the MiSeq paired-end Illumina platform adapted for 2 × 250 bp paired-end chemistry. Sequence reads were then processed, aligned, and categorized using the DADA2 (v1.8) pipeline to infer exact amplicon sequence variants (SVs) from amplicon data [35]. Briefly, sequence reads were filtered (reads truncated after a quality score of ≤2 and forward/reverse reads truncated after 155/110 bases, respectively) and trimmed (10 bases off 5′ end of reverse reads) using optimized parameter settings as recommended. Next, sequence reads were de-replicated, de-noised, and merged using DADA2 default parameters with read recovery rates ranging from 83.9% to 94.5%. Taxonomy was assigned to SVs using a customized database consisting of the SILVA non-redundant v132 training set and a previously established honey bee-specific seed alignment of 276 unique representatives [36]. Raw sequence reads were uploaded to the NCBI Sequence Read Archive and are accessible under BioProject ID PRJNA525184.

### In vitro inhibition assays for *P. larvae*

Vegetative *P. larvae* cells were cultivated via aerobic growth in MY media at 37 °C for 48 h, followed by a 1:50 sub-culturing step, and then harvested during mid log-phase. Bacterial suspensions were then adjusted to OD_600_ = 0.75 and spread over freshly prepared MY agar plates (300 µL) as described previously [37]. Lactobacilli strains of interest were grown to stationary phase under their optimal growth conditions (described above). Subsequently, cells were gently centrifuged at 4500×*g* and then washed twice in 0.01 M PBS, followed by resuspension in 0.01 M PBS at an adjusted concentration of 1 × 10^9^ cells/mL. The resultant suspensions (20 µL) were spotted onto sterile filter disks (7 mm diameter; Whatman) and placed onto MY plates freshly spread with *P. larvae*. Plates were incubated in microaerophilic conditions under 5% CO_2_ at 37 °C and zones of inhibition measured after 48 h. Sterile 0.01 M PBS served as a negative control, which showed no effect on *P. larvae* growth. All antibiotic control disks (diameter = 7 mm) contained 30 µg of either tetracycline, doxycycline, or oxytetracycline hydrochloride (Oxoid; Thermo Scientific).

Inhibition of *P. larvae* growth in solution was tested with the incubation of cell-free supernatant (CFS) from Lp39, LGR-1, and LkBR-1. All bacterial strains tested were cultured in MY (with the addition of 10 g/L d-fructose for LkBR-1; blank vehicle controls for this media failed to demonstrate any inhibitory properties on *P. larvae*) under each of their aforementioned optimal growth conditions, and then were harvested in stationary phase and adjusted to 1 × 10^9^ CFU/mL. Subsequently, bacterial suspensions were 0.2 µM filtered-sterilized to obtain CFSs, which were then pH-adjusted (pH = 6.2; original pH of media) to eliminate any non-specific influence that pH differences may have on *P. larvae* growth. Vegetative *P. larvae* cells grown aerobically at 37 °C in MY were obtained in mid log-phase as above, and then diluted to OD_600_ = 0.1 in fresh MY media with the addition of 12.5% CFS (v/v) or 12.5% 0.2 µM filtered-sterilized MY vehicle. Suspensions were then added to a 96-well U-bottom plate in 200 µL aliquots in technical triplicate prior to sealing of wells with optically clear adhesive films. Plates were incubated at 37 °C with 150 RPM orbital shaking for 48 h with OD_600_ measurements taken every 30 min using a BioTek microplate reader.

### Fluorescent-based bacterial cell viability assays

Log-phase harvested *P. larvae* and stationary-phase Lp39 were gently centrifuged at 4500×*g* for 10 min, washed twice in 0.01 M PBS, and re-suspended in glucose-supplemented Krebs–Ringer solution (120 mM NaCl, 4.9 mM KCl, 1.2 mM MgSO_4_, 1.7 mM KH_2_PO_4_, 8.3 mM Na_2_HPO_4_, and 10 mM glucose, pH 7.3). Co-incubation of Lp39 and *P. larvae* was 1 h in duration and performed with 1 × 10^7^ CFU/mL of each bacteria. Following incubation, bacterial cells were stained using the ViaGram Red + Bacterial Gram Stain and Viability Kit (Invitrogen) according to manufacturer's recommendations. Subsequently, samples were sealed under a coverslip and visualized using the 60× oil-immersion lens on a Nikon Eclipse T*i*2-A confocal microscope. Bacterial cells were identified on the basis of their differential morphology, with long rod-shaped bacterium representing *P. larvae* and short rod-shaped bacteria representing Lp39.

### Infection assays using laboratory-reared honey bee larvae

First-instar honey bee larvae were removed from ten nearby hives exhibiting no sign of disease using a Chinese grafting tool, placed in 6-well tissue culture plates containing 2.5 mL of RJb1 media (50% (w/v) royal jelly, 0.9% (w/v) yeast extract, 5.1% (w/v) d-glucose, and 5.1% (w/v) d-fructose), and then were transported to laboratory conditions in an insulated container maintained at 37 °C. Individuals were then randomized without regard for their colony of origin, pooled into groups of *n* = 40, separated into 6-well tissue culture plates containing 2.5 mL of RJb1 media, and orally supplemented either LX3 (1 × 10^7^ CFU/mL of each Lp39, LGR-1, and LkBR-1) or vehicle (0.01 M PBS) for 24 h prior to subsequent infection. Second-instar larvae were then transferred to fresh individual wells in a 96-well flat-bottom tissue culture plate containing 25 µL of RJb2 media (50% (w/v) royal jelly, 1.3% (w/v) yeast extract, 6.4% (w/v) d-glucose, and 6.4% (w/v) d-fructose) with the addition of 1 × 10^4^ spores of *P. larvae* or vehicle (sterile ddH_2_O) as described previously [17]. On day 1 post-infection, honey bee larvae were fed fresh RJb3 media (50% (w/v) royal jelly, 1.7% (w/v) yeast extract, 7.7% (w/v) d-glucose, and 7.7% (w/v) d-fructose) for the remainder of the experiment with incremental increases in volume of 10 µL/day with a starting diet of 25 µL on day 1. Subsequently, larvae were monitored for survival every 24 h via gentle surface agitation using a sterile pipette tip. Individuals were considered dead on the basis of an absent response to physical stimuli and the sustained lack of movement or respiration [17]. Dead larvae were immediately removed from their well.

### TRIzol-based RNA extraction and qPCR for host gene expression

In vitro-reared honey bee larvae were surface-sterilized using 0.25% sodium hypochlorite. RNA was then extracted from whole larvae using 700 µL of TRIzol (Invitrogen) following manufacturer’s instructions. Quality of RNA was evaluated using a microvolume spectrophotometer (DS-11 Spectrophotometer; DeNovix) and determined to have A260/280 absorbances ratios between 1.9 and 2.2. cDNA was synthesized from 1500 ng of total RNA using a High-Capacity cDNA Reverse Transcription Kit following manufacturer’s instructions (Applied Biosystems, catalogue number: 4368813).

Previously established oligonucleotide primers [38, 39] were used for qPCR reactions and are listed in Supplementary Table 2. Preliminary experiments identified honey bee *alpha-tubulin* (XM_391936) to be most stably expressed (compared to *ribosomal protein S5* [XM_624081], *microsomal glutathione-S-transferase* [XM_394313], and *UDP-glucuronyltransferase* [XM_392727]) endogenous control under our specific set of experimental conditions, and thus was chosen as the internal standard for normalization as per MIQE guidelines [40]. cDNA was diluted tenfold and used for qPCR reactions with the Power SYBR Green kit (Applied Biosystems) as previously described [19]. All qPCR reactions were performed in DNase- and RNase-free 384-well microplates using a Quant Studio 5 Real-Time PCR System (Applied Biosystems) and analyzed with associated software. Relative gene expression was calculated using the 2^−ΔΔ Ct^ method [41]. PCR amplification was confirmed via melt-curve dissociation analyses to verify expected product and check for non-specific amplification.

### Simultaneous extraction of DNA following RNA extraction

DNA was back-extracted from the TRIzol homogenates of laboratory-reared honey bee larvae (described above) using a back-extraction buffer (BEB) consisting of 4 M guanidine thiocyanate, 50 mM sodium citrate, and 1 M Tris base as previously described [42]. Samples were diluted and then used for qPCR as described above to assess the microbial loads of major phylotypes in laboratory-reared honey bee larvae during *P. larvae* infection.

## Results

### Retrospective analysis of BioPatty supplementation following natural AFB outbreak

**Fig. 1:**
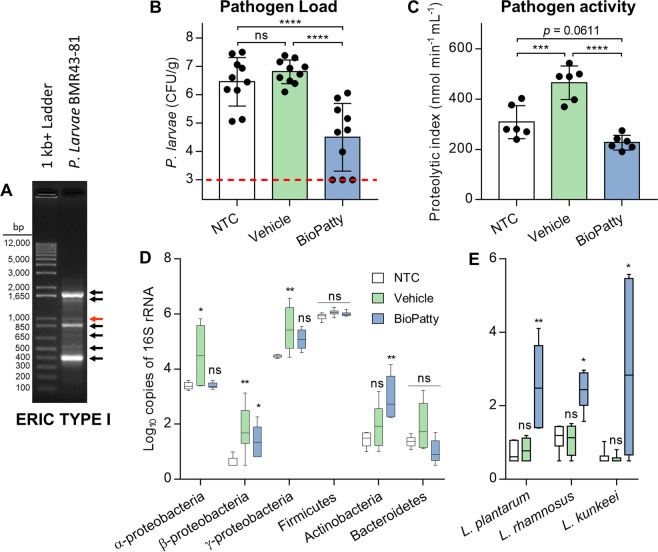
Retrospective analysis of BioPatty supplementation following natural AFB outbreak. **a** Molecular identification of *Paenibacillus larvae* BMR43-81 by rep-PCR using ERIC primers. Red arrow = 970 bp confirmation band for *P. larvae* subsp. *larvae*. Black arrows = characteristic banding pattern for previously established ERIC subtype I profile. **b** Pathogen load of whole honey bee larvae from inner brood frames of experimental hives was determined by plating extracted homogenates on MY agar media. Colony forming units (CFU) obtained represent the mean ± standard deviation (one-way ANOVA with Tukey’s multiple comparisons) of *n* = 10 pooled larval samples for each treatment group (three larvae per pooled sample). **c** Pathogen activity of whole honey bee larvae from inner brood frames of experimental hives was determined via a modified Holst milk test clearance assay. Mean casein hydrolysis ± standard deviation (one-way ANOVA with Tukey’s multiple comparisons) of *n* = 6 pooled larval samples for each treatment group (three larvae per pooled sample) with triplicate technical repeats are shown. **d**, **e** qPCR-based quantification of dominant microbiota phylotypes and supplemental lactobacilli across treatment groups. Data represents the median (line in box), IQR (box), and minimum/maximum (whiskers) of *n* = 6 pooled larval samples for each treatment group (three larvae per pooled sample) with duplicate technical repeats. Statistical comparisons shown for one-way ANOVA (dominant microbiota phylotypes) and Kruskal–Wallis (supplemental lactobacilli) tests with Dunnett’s and Dunn’s multiple comparisons, respectively. ns = not significant, *P < 0.05, **P < 0.01, ***P < 0.001, and ****P < 0.0001

After 12 days of experimentation, classical signs of AFB were detected using the qualitative in-field “rope-test” [27]. This was confirmed by isolation of a non-pigmented strain of *P. larvae* from brood samples exhibiting signs of disease. Molecular identification via 16S rRNA gene sequencing, followed by a BLAST search against the GenBank Bacteria and Archaea 16S ribosomal RNA sequences database (NCBI), demonstrated the isolate to most closely match *P. larvae* strain DSM 7030 (Query cover = 99%, *E*-value = 0.0, and Identity = 99.45%; NR_042947.1). Furthermore, ERIC-subtyping of the *P. larvae* isolate using rep-PCR demonstrated a banding pattern (Fig. 1a) that matched well with the previously characterized *P. larvae* ERIC subtype I [43].

To determine differences in larval pathogen load between treatment groups, *P. larvae* abundances were enumerated in third- to fifth-instar larvae using a cultured-based method [27]. Larvae from the BioPatty-supplemented group exhibited significantly lower pathogen loads in comparison to NTC and vehicle-supplemented groups (one-way ANOVA with Tukey’s multiple comparisons, *P* < 0.0001 for both), with no observable differences between the latter two groups (Fig. 1b).

Larval samples from vehicle-supplemented groups were shown to have a significantly higher proteolytic index than samples from NTC and BioPatty-supplemented groups (one-way ANOVA with Tukey’s multiple comparisons, *P* = 0.0006 and *P* < 0.0001, respectively; Fig. 1c). A trend towards decreased proteolytic activity was observed in the BioPatty treatment group relative to NTC (one-way ANOVA with Tukey’s multiple comparisons, *P* = 0.0611; Fig. 1c).

Using a qPCR-based approach to enumerate low levels of bacteria in honey bee larvae [32], the six major phylotypes commonly associated with the microbiota of honey bees were measured. Larval samples from the vehicle-supplemented group displayed significantly higher levels of Alphaproteobacteria, Betaproteobacteria, and Gammaproteobacteria (one-way ANOVA with Dunnett’s multiple comparisons, *P* = 0.0222, *P* = 0.0084, *P* = 0.0069, respectively) compared to the NTC group (Fig. 1d). BioPatty-supplemented larvae, by contrast, had significantly higher levels of Actinobacteria and Betaproteobacteria (one-way ANOVA with Dunnett’s multiple comparisons, *P* = 0.0026, and *P* = 0.0431, respectively) compared to the NTC group, but no differences were found in Alphaproteobacteria and Gammaproteobacteria loads (Fig. 1d). Moreover, using species-specific primers, it was found that larval samples from the BioPatty group had significantly higher levels of *L. plantarum*, *L. rhamnosus*, *and L. kunkeei* (Kruskal–Wallis test with Dunn’s multiple comparisons, *P* = 0.0080, *P* = 0.0135, and *P* = 0.0417, respectively) compared to the NTC group on day 12 of the field trial (Fig. 1e).

### Total bacterial loads and 16S rRNA sequencing of the adult honey bee gut microbiota during an AFB outbreak under natural field conditions

**Fig. 2:**
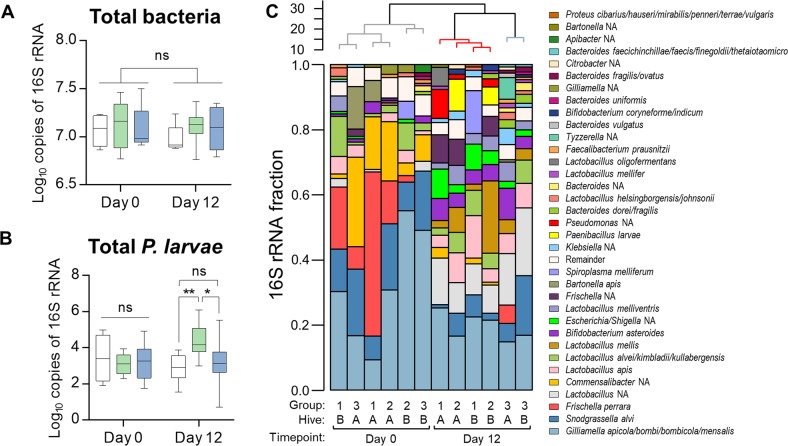
Total bacterial loads and 16S rRNA sequencing of the adult honey bee gut microbiota during an AFB outbreak under natural field conditions. qPCR-based quantification of **a** total gut bacteria and **b** total *P. larvae* loads in surface-sterilized adult nurse bees. Data represent the median (line in box) and minimum/maximum (whiskers) of eight adult gut samples in each treatment group with duplicate technical repeats performed. Statistical analysis shown for two-way ANOVA with Tukey’s multiple comparisons. ns = not significant, *P < 0.05, **P < 0.01. **c** Bar plots represent the gut microbiota compositions of a single bee from each of their respective treatment groups as determined by sequencing of the V4 region of the bacterial 16S rRNA gene. Taxonomy was assigned using a custom database created by combining a previously established dataset of bee-associated 16S rRNA gene sequences with the SILVA NR v132 training set. Group 1 = no treatment, Group 2 = vehicle pollen patty only, and Group 3 = BioPatty. Hierarchal clustering of samples is shown in the dendrogram above the bar plot and was calculated using the ward.D method and “hclust” function in R. Cluster 1 = grey, Cluster 2 = red, Cluster 3 = blue

To further examine potential polymicrobial interactions and dynamic changes that occur in the bacterial communities associated with honey bees during the AFB outbreak, 16S rRNA gene sequencing was performed on the gut microbiota of adult worker bees. Nurse-aged adult bees were chosen for examination based on their close association with brood and previous reports demonstrating them to be good estimators of overall hive microbial diversity [12]. After omitting control samples, the total nurse bee microbiota dataset contained 579,789 reads, ranging from 24,352 to 81,014 reads per sample. An average of 8.96% of total reads were removed from each sample following quality assurance measures using the DADA2 pipeline [35], leaving a total of 527,824 filtered reads. Taxonomy was assigned to SVs using a custom-designed classification database consisting of the SILVA non-redundant v132 training set and a honey-bee specific database of high-quality reference sequences [36]. SVs identified as *Wolbachia* spp. or chloroplasts were removed. After implementing a 1% abundance cut off, a total of 112 unique SVs remained. A bar plot and dendrogram visually representing the relative proportions of taxa in the samples is provided in Fig. 2c. These results are consistent with past surveys demonstrating a simple and distinctive community profile in the adult honey bee gut microbiota [30, 32].

The dendrogram in Fig. 2c shows distinct clustering of samples based on time-point (day 0 vs day 12) and between samples on day 12 (NTC and vehicle-treated samples vs BioPatty samples) based on Aitchison distances, a suitable metric for the analysis of compositional data [44, 45]. No significant differences in total bacteria loads existed between any of the treatment groups at any time-point during the field trial, based on qPCR-based quantification of total bacteria load using universal 16S rRNA primers and honey bee *β-actin* as a loading control (Fig. 2a). However, *P. larvae* levels in nurse bees from vehicle-treated hives were significantly higher than in NTC and BioPatty treatment groups on day 12 (two-way ANOVA with Tukey’s multiple comparisons, *P* = 0.0053 and *P* = 0.0245, respectively; Fig. 2b).

### Exploratory comparison of the gut microbiota in adult nurse bees during AFB outbreak

**Fig. 3:**
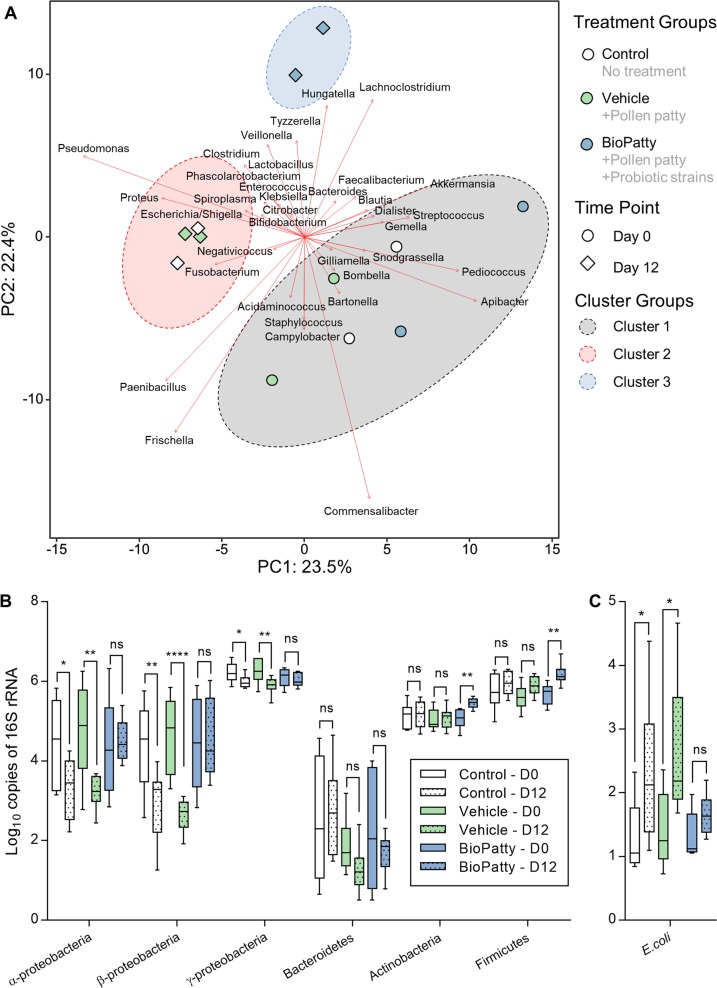
Exploratory comparison of the gut microbiota in adult nurse bees during AFB outbreak. **a** Principle Component Analysis (PCA) plot of adult honey bee gut microbiota samples. Sequence variants were collapsed at genus-level identification, with CLR-transformed Aitchison distances used as input values for PCA analysis. Distance between individual samples (points) represents differences in microbiota composition, with 45.9% of variance explained by the first two principle components shown. Strengths of association for genera are depicted by the length of the red arrows. Clustering of samples was determined using the “*k*-means” function in R. **b**, **c** qPCR-based quantification of dominant microbiota phylotypes and *Escherichia coli* in adult nurse bee gut samples. Data represent the median (line in box), IQR (box), and minimum/maximum (whiskers) of ten individual gut samples with duplicate technical repeats. Statistical analysis shown for one-way ANOVA with Benjamini and Hochberg corrected multiple comparisons. ns = not significant, *P < 0.05, **P < 0.01, ***P < 0.001

Using the 112 unique SVs identified, samples were centred log ratio (CLR) transformed to generate Aitchison distances, which were subsequently used to perform a principal component analysis on the nurse bee microbiota dataset (Fig. 3a). Principal components 1 and 2 explain 45.9% of the total variance in the microbiota composition between individual samples (Fig. 3a). Additionally, *k*-means clustering was used to partition samples into distinctive groups that had similar microbiota compositions. Three distinctive clusters were calculated and shown to be associated with both experimental time-point and treatment. The largest influencers were identified as species from *Apibacter*, *Commensalibacter*, *Frischella*, *Paenibacillus*, and *Pseudomonas* (strength of association depicted by red arrows; Fig. 3a).

To further validate the 16S rRNA gene sequencing dataset, bacteria in the gut microbiota of adult nurse bees were quantified by qPCR using established phylotype-specific primers [30–32]. NTC and vehicle-supplemented groups were found to have significantly less Alphaproteobacteria (one-way ANOVA with Benjamini and Hochberg multiple comparisons, *P* = 0.0190*, P* = 0.0019), Betaproteobacteria (one-way ANOVA with Benjamini and Hochberg multiple comparisons, *P* = 0.0046, *P* = 0.0001), and Gammaproteobacteria (one-way ANOVA with Benjamini and Hochberg multiple comparisons, *P* = 0.0151, *P* = 0.0029) on day 12 (post-AFB detection) in comparison to day 0 (pre-AFB detection; Fig. 3b). BioPatty-treated groups had higher levels of Actinobacteria and Firmicutes (one-way ANOVA with Benjamini and Hochberg multiple comparisons, *P* = 0.0083 and *P* = 0.0066, respectively) on day 12 compared to day 0 (Fig. 3b). Based on observations from the compositional dataset (Fig. 2c and 3a), *Escherichia coli* was quantified via qPCR using species-specific primers [33]. Absolute abundance of *E. coli* in adult nurses was found to be significantly higher on day 12 compared to day 0 for NTC and vehicle-supplemented groups but not the BioPatty-supplemented group (one-way ANOVA with Benjamini and Hochberg multiple comparisons, *P* = 0.0351, *P* = 0.0217, *P* = 0.7302, respectively; Fig. 3c).

### In vitro growth and cell viability of *P. larvae* is reduced by Lp39

**Fig. 4:**
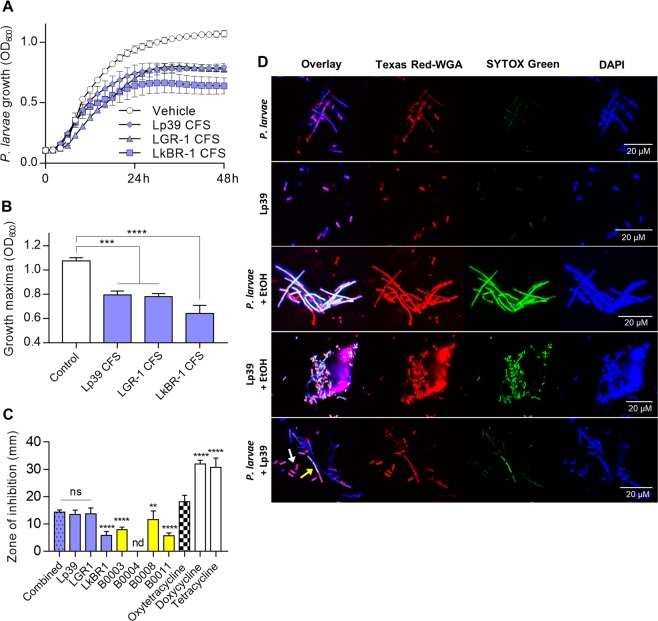
In vitro growth and cell viability of *Paenibacillus larvae* is reduced by Lp39. **a** Growth curves of *P. larvae* in MY media supplemented with cell-free supernatant from lactobacilli strains of interest. **b** Percent maximal growth was determined from growth curve data (OD_600_) at 48 h using the area under the curve for *P. larvae* grown in MY media supplemented with CFS from the specified lactobacilli. Data are depicted as means ± standard deviation (one-way ANOVA with Dunnett’s multiple comparisons) of *n* = 3 biological replicates performed with duplicate technical repeats. **c** Zone of inhibition measurements represent the mean ± standard deviation radius clearance (minus the disk) on a *P. larvae* lawn grown on MY agar. Experiments were performed in biological triplicate (*n* = 3 for each group) with technical duplicates. Statistical analysis is shown for one-way ANOVA with Dunnett’s multiple comparisons made against 30 µg of oxytetracycline. *Enterobacter hormaechei* B0003, *Paenibacillus illinoisensis* B0004, *Hafnia paralvei* B0008, and *Lactobacillus apis* B0011 represent isolates previously obtained from a healthy hive. **d** Lp39 (short rod-shaped) and *P. larvae* (long rod-shaped) were incubated in nutrient-limited media for 60 min and subsequently stained with cell-permeable (4′,6-diamidino-2-phenylindole; DAPI) and non-permeable (SYTOX Green) nucleic acid markers, as well as Texas Red-WGA that selectively binds to the surface of gram-positive bacteria. Cells were visualized using a Nikon Eclipse Ti2 confocal microscope. Increased uptake of SYTOX Green indicates reduced cell viability based on plasma membrane integrity. Yellow arrow points to *P. larvae*, white arrow points to Lp39. Bacterial cells that were incubated with 70% ethanol (EtOH) served as a positive control to validate the assay. Scale bar = 20 µM. ns = not significant, **P < 0.01, ***P < 0.001, ****P < 0.0001

CFS from stationary-phase Lp39, LGR 1, and LkBR-1 grown in MY media were tested for their ability to inhibit *P. larvae* growth in solution. Following incubation with 12.5% CFS, time-coursed measurement of *P. larvae* growth demonstrated that all lactobacilli strains negatively affected the growth maxima of *P. larvae* in solution (Fig. 4a, b). Using standard plate-based zone of inhibition assays [13], we tested the *P. larvae*-inhibiting properties of several common antibiotics, specific lactobacilli strains of interest, and previously derived honey bee isolates. All antibiotics and bacteria, except *Paenibacillus illinoisensis* B0004, showed some level of inhibition against *P. larvae* on solid surface growth media (Fig. 4c). Lp39, LGR-1, and the combination of Lp39, LGR-1, and LkBR-1 were as efficient as the guideline recommended antibiotic, oxytetracycline, in their ability to inhibit *P. larvae* (Fig. 4c). Other tetracycline-related antibiotics, including tetracycline itself and doxycycline, were significantly more effective at inhibiting *P. larvae* than oxytetracycline (one-way ANOVA with Dunnett’s multiple comparisons, *P* < 0.0001 for both; Fig. 4c).

These findings compliment numerous studies demonstrating the inhibitory properties of various lactobacilli on *P. larvae* both in vitro [37] and in vivo [46, 47]; however, they did not explain whether the tested lactobacilli can kill *P. larvae* cells or simply inhibit their growth similar to bacteriostatic antibiotics. A fluorescent-based cytotoxicity assay on Lp39 and *P. larvae* cells demonstrated uptake of SYTOX Green in *P. larvae* cells (long and rod-shaped) but not Lp39 cells (short and rod-shaped) during co-incubation for 1 h in a glucose-supplemented physiological buffer (Fig. 4d).

### Prophylactic supplementation of Lp39, LGR-1, and LkBR-1 (LX3) improves survival during natural *P. larvae* infection

**Fig. 5:**
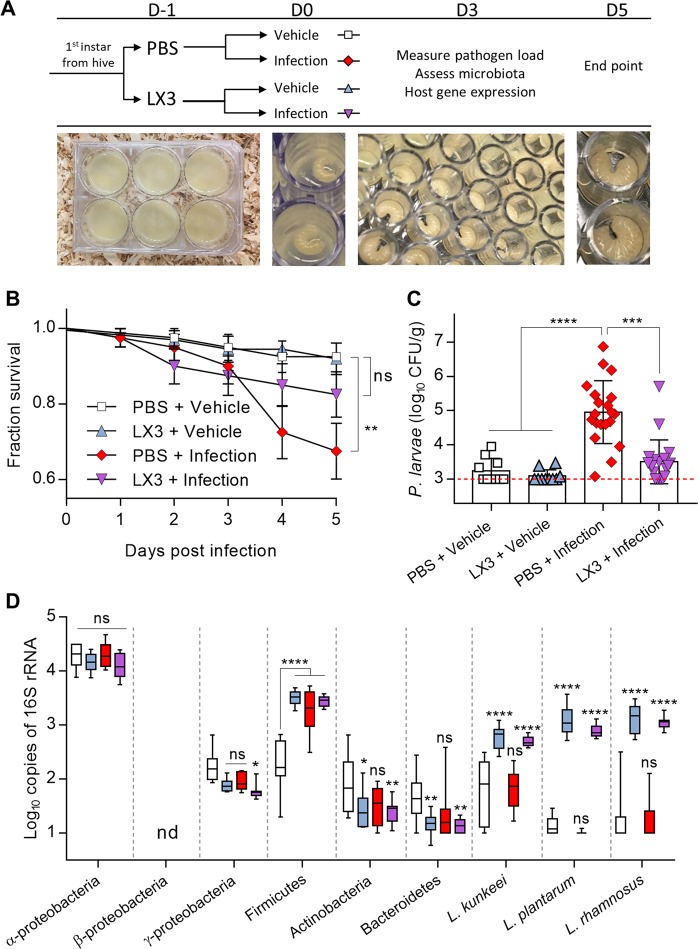
Prophylactic supplementation of Lp39, LGR-1, and LkBR-1 (LX3) improves survival during natural *P. larvae* infection. **a** Schematic diagram illustrating the experimental design for laboratory rearing of honey bee larvae and infection timeline. **b** Survival curves for laboratory-reared second-instar honey bee larvae that were subjected to natural infection with *P. larvae* BMR43-81 with or without 24 h pre-supplementation with LX3 delivered orally (10^7^ CFU/mL for each strain). All statistical symbols are representative of comparisons made to respective vehicle control groups using the log-rank (Mantel–Cox; *n* = 40 individuals for each treatment group) test. **c** Pathogen load of whole honey bee larvae at day 3 post infection was determined by plating extracted homogenates on MY agar media. Colony forming units (CFU) are represented by the median with 95% confidence intervals (Kruskal–Wallis test with Dunn’s multiple comparisons) shown for 10–20 individual larvae in each group as depicted by symbols on the graph. **d** qPCR-based quantification of dominant microbiota phylotypes and supplemental lactobacilli across treatment groups at day 3 post infection. Data represents the median (line in box) and minimum/maximum (whiskers) of eight individual larval samples per treatment group. Statistical analysis is shown for two-way ANOVA with Tukey’s multiple comparisons made against the non-infected PBS control group. nd = not detectable, ns = not significant, *P < 0.05, **P < 0.01, ***P < 0.001, and ****P < 0.0001

LX3 supplementation significantly improved overall survival (log-rank (Mantel–Cox), *χ*^2^ = 11.79, *P* = 0.0081) and reduced early time-point deaths (Gehan–Breslow–Wilcoxon test, *χ*^2^ = 4.462, *P* = 0.0347) during infection compared to PBS-supplemented vehicles (Fig. 5b). In addition, LX3-supplemented honey bee larvae exhibited significantly reduced levels of *P. larvae* (Kruskal–Wallis test with Dunn’s multiple comparisons, *P* = 0.0005) at 3 days post-infection compared to PBS-supplemented individuals (Fig. 5c).

Using a qPCR-based approach to enumerate low levels of indigenous bacteria in the microbiota of in vitro-reared honey bee larvae during infection, the same six major phylotypes previously assessed in day 12 larval samples from our field-trials were measured (Fig. 1d). Under laboratory-controlled conditions, *P. larvae* infection had no significant effect on any of the phylotypes tested when compared with non-infected controls on day 3 post-infection. Infected honey bee larvae supplemented with LX3 demonstrated significantly lower levels of Gammaproteobacteria and Bacteroidetes (two-way ANOVA with Tukey’s multiple comparisons, *P* = 0.0195 and *P* = 0.0046, respectively) compared to non-infected PBS supplemented controls (Fig. 5d). Consistent with field data, the use of species-specific primers showed that both infected and non-infected larvae supplemented with LX3 had significantly higher levels of *L. plantarum*, *L. rhamnosus*, and *L. kunkeei* compared to infected and non-infected PBS-supplemented larvae at 96 h following initial supplementation (Fig. 5d).

### LX3 increases immune-related gene expression during *P. larvae* infection

**Fig. 6:**
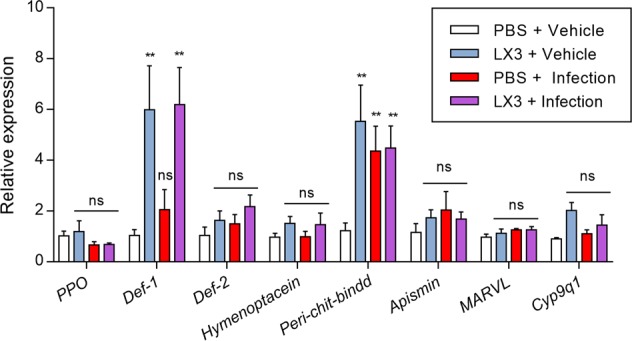
LX3 increases immune-related gene expression during *P. larvae* infection. First-instar honey bee larvae were orally supplemented with LX3 (*Lactobacillus plantarum* Lp39, *Lactobacillus rhamnosus* GR-1, and *Lactobacillus kunkeei* BR-1) or vehicle for 24 h, followed by inoculation with 10^4^ spores of *P. larvae*. Expression of immune-related and cellular-response genes were quantified via RT-qPCR at 72 h post-infection. All statistical comparisons are relative to the non-infected PBS control group and calculated with raw ΔΔCt values. Mean ± standard deviation (one-way ANOVA with Holm–Sidak’s multiple comparisons) of six larvae per treatment group with technical duplicate repeats are shown. ns = not significant, *P < 0.05, **P < 0.01, and ***P < 0.001

Increased expression of key immune-related genes has been shown to parallel very closely with the ability of honey bee larvae to resist *P. larvae* infection [48]. Here, prophylactic supplementation with LX3 (24 h) significantly upregulated *Def-1* and *Pcbd* at 72 h post-infection compared to PBS-supplemented controls (one-way ANOVA with Holm–Sidak’s multiple comparisons, *P* = 0.0079 and *P* = 0.0110, respectively; Fig. 6). LX3 administration alone also significantly increased expression of these genes in the absence of *P. larvae* inoculation (one-way ANOVA with Holm–Sidak’s multiple comparisons, *P* = 0.0146 and *P* = 0.0106, respectively), compared to PBS-supplemented controls. No changes were observed in *Ppo*, *Def-2*, *Hymenoptacein*, or *Apismin* (Fig. 6).

## Discussion

This study demonstrated the utilization of lactobacilli, via in-hive BioPatty supplementation, to improve honey bee survival and hive resilience against *P. larvae*—the spore-forming bacterium responsible for AFB. Notably, endpoint measurements following the 12-day field trial in which the AFB outbreak inadvertently occurred showed pathogen load and activity to be significantly lower in honey bee larvae treated with the BioPatty, compared to vehicle controls receiving only the base pollen patty ingredients (Fig. 1b–d). These findings were further validated in vitro by performing infection survival assays on laboratory-reared honey bees. Under these controlled conditions, prophylactic supplementation of LX3 (containing Lp39, LGR-1, and LkBR-1 strains of lactobacilli present in the BioPatty) significantly reduced pathogen load and markers of disease, increased survival, and upregulated gene expression of key antimicrobial peptides involved in host defenses against *P. larvae* (Figs. 4b, c,  5). These results expand on previous work demonstrating Lp39-mediated priming of innate immunity in *D. melanogaster* [19] and corroborate the findings that *L. kunkeei* [49] and other lactic acid bacteria [37, 47] can inhibit *P. larvae*. Our findings are contrary to a recent report that lactic acid bacteria have no effect on *P. larvae* at the colony level [50]. Though, the discrepancies might be explained by the fact that Stephan et al. [50] administered their supplemental bacteria using a 15% sucrose solution vehicle—likely resulting in a stark reduction of bacterial cell viability given this medium is known to induce severe osmotic stress in lactic acid bacteria [51]. This may also explain why the supplemented bacteria failed to demonstrate any biological activity against *P. larvae* in Stephan et al. [50] and suggests that delivery of viable bacteria to the hive is of key importance.

A significant increase was found in proteolytic activity of homogenized larvae (common marker for pathogen activity and in-field detection of AFB) from vehicle pollen patty-supplemented hives compared to NTC hives (Fig. 1c)—despite no differences in pathogen load (Fig. 1b). These results, alongside a significant increase in Alphaproteobacteria, Betaproteobacteria, and Gammaproteobacteria in honey bee larvae (Fig. 1d), raise the question of whether pollen patty supplementation per se might stimulate the growth of unwanted organisms. Eliminating the possibility of these bacteria being saprophytic secondary invaders following *P. larvae* infection, no changes were observed in the abundance of these bacteria between infected and non-infected honey bee larvae reared in vitro (Fig. 5d). These results should be cautiously interpreted as in vitro rearing of honey bee larvae cannot perfectly emulate the highly complex microbial dynamics, nor the organized social feeding behaviours that are present in a hive. However, proteobacterial “blooming” in humans and mice is considered a signature of dysbiosis attributable to excess dietary protein, an unstable microbial community structure, and/or dysregulated immunity [52]. Thus, increased growth of proteobacteria resulting from excess protein in the vehicle pollen patties could explain these discordant results given these bacteria have more than a ten-fold enrichment in proteolytic enzymes compared to other phyla commonly associated with animals [53].

There is limited evidence to support a causal linkage between usage of pollen patties and incidence of AFB outbreak; however, recent reports have demonstrated that commercial pollen substitutes can significantly increase *Nosema* spp. (microsporidian parasites) abundances [54], and that protein-supplemented hives have higher titres of Black Queen Cell Virus and greater queen losses compared to natural forage-supplemented hives [55]. Here, we demonstrate that nurse bees (young adult workers in close association with brood and good estimators of overall hive microbial diversity) exhibited a distinctive shift in their gut microbiota during the AFB outbreak irrespective of treatment group (Fig. 2c). While a reduction in the abundance of core phylotype members Alphaproteobacteria, Betaproteobacteria, and Gammaproteobacteria was observed in NTC and vehicle-supplemented groups, an increase in Actinobacteria and Firmicutes was found in the BioPatty-supplemented group (Fig. 3b). Despite these differential shifts in microbiota composition, only vehicle-supplemented nurse bees experienced significantly higher levels of *P. larvae* on day 12 (Fig. 2b). Together with the findings of similar *P. larvae* loads in NTC and vehicle-supplemented honey bee larvae (Fig. 1b), these results suggest that standard pollen patties are likely not the primary initiators of disease but may increase overall hive burden through exacerbating *P. larvae* growth in asymptomatic carriers. Importantly, no detectable changes in pathogen burden were observed in adult nurse bees from the BioPatty-supplemented group, and larval samples from this group had significantly lower levels of *P. larvae* on day 12—further substantiating the benefits of infusing pollen patties with lactic acid bacteria [26, 56–58].

In corroboration with compositional data (Figs. 2c and 3a), absolute abundance of *E. coli* in adult nurse bees from NTC and vehicle-supplemented groups was significantly increased (Fig. 3c), suggesting a partial substitution of core microbiota members with opportunistic pathogens during the AFB outbreak. Supporting these observations further, it’s been shown that core microbiota members, such as *Snodgrassella alvi* (Betaproteobacteria), help to suppress the growth of *E. coli* in honey bees [59]. Interestingly, the protein-catabolizing enzyme xanthine dehydrogenase secreted by *Escherichia* spp. [60] can facilitate oxidative metabolism of purines to uric acid—a known requirement for *P. larvae* germination [61]. Future studies will be required, however, to determine whether AFB outbreaks arise through a microbiota-driven process similar to other bee diseases [59, 62–64] or if the infection itself is the root cause of microbiota alterations.

The factors affecting honey bee gut dysbiosis remain largely unknown [65], though innate immune response [66] and environmental landscape [67] are thought to be primary influencers. Thus, the ability of *Lactobacillus* spp. to modulate honey bee immunity could explain some of the differential microbiota changes and lower *E. coli* loads observed in adult nurse bees from BioPatty-supplemented hives (Fig. 3c). Moreover, *Lactobacillus* spp. are largely enriched in uric acid catabolism enzymes including uricase (EC 1.7.3.3), allantoinase (EC 3.5.2.5), and allantoicase (EC 3.5.3.4) [68]. Here, we demonstrated Lp39 (uniquely possessing tyrosine decarboxylase (EC 4.1.1.25); an enzyme capable of breaking down tyrosine—another essential germinant of *P. larvae*) was able to induce cytotoxic effects against *P. larvae* cells through an uncharacterized mechanism (Fig. 4d). Furthermore, prophylactic supplementation of LX3 significantly improved survival and decreased *P. larvae* loads in experimentally infected laboratory-reared honey bee larvae (Fig. 5b, c). These in vitro findings support observations from our hive experiments and suggest that the lactobacilli strains tested in this study could offer a distinct advantage over antibiotics, such as oxytetracycline, through both directly inhibiting germination and actively reducing cell viability of *P. larvae*.

Another way in which honey bees rely on beneficial bacteria for protection against infectious disease is through immune modulation. Infected honey bee larvae that were prophylactically supplemented with LX3 demonstrated significant upregulation in *Def-1* and *Pcbd* (Fig. 6)—which encode an antimicrobial peptide and peritrophic matrix-related protein important to resisting *P. larvae* infection, respectively [6, 69]. Furthermore, LX3 supplementation alone strongly upregulated *Def-1* (an isoform of honey bee *Defensin* primarily involved in social immunity) independent of infection with *P. larvae* but had no effect on *Def-2* (an isoform responsible individual immunity) expression under any of the conditions tested [70]. As in previous studies [38, 69], infection with *P. larvae* failed to elicit an observable change in the gene expression of other major honey bee antimicrobial peptides (Fig. 6). Together, these results suggest hive administration of lactobacilli may support broad-spectrum protection towards infectious disease through priming the innate immune system. As an aside, *Ppo* expression showed a trend towards downregulation in *P. larvae*-infected groups during the in vitro larval infection assays. *Ppo* is believed to be a suitable biomarker for hemocyte abundance based on the findings that its expression is directly correlated with hemocyte counts during parasitism by *Varroa destructor*—a deleterious mite parasite that reduces hemocyte concentrations by feeding on the fat body of honey bees [71, 72]. The possibility of a synergistic interaction between *P. larvae* and *V. destructor* on immunity supports the growing theory that multi-faceted pest and pathogen networks are at the centre of global bee decline [73].

From an ecological perspective, controlling the spread of enzootic pathogens in managed bees is critical to maintaining wild pollinators as well, which are suspected to be declining in concert as a result of interspecies pathogen transmission within the pollinator assemblage [74]. Addressing this issue without routine usage of antibiotics, which pollute the environment and lead to accumulation of antibiotic-resistance genes [25], will be paramount in the ongoing fight to save pollinators.

In summary, although this was only a single field trial study, the serendipitous nature in which AFB occurred facilitated the identification of several unique factors that may help better understand the aetiology of *P. larvae*—particularly aspects influencing its highly elusive germination cycle in the hive. Observations from the field trial were well supported by laboratory-controlled experiments, which further demonstrated that the triple-strain lactobacilli consortium could: (i) improve honey bee survival towards *P. larvae* infection, (ii) directly inhibit *P. larvae* cells in vitro, and (iii) beneficially modulate innate immunity and other host-response genes during experimental infection. Although the lactobacilli tested in this study were shown to be beneficial under infectious conditions, further studies will be needed to determine their long-term impacts on healthy honey bee hives.

## Supplementary information

Supplementary Table 1

Supplementary Table 2
